# Validity, reliability, and responsiveness of daily monitoring visual analog scales in MASK‐air®

**DOI:** 10.1002/clt2.12062

**Published:** 2021-09-19

**Authors:** Bernardo Sousa‐Pinto, Patrik Eklund, Oliver Pfaar, Ludger Klimek, Torsten Zuberbier, Wienczyslawa Czarlewski, Annabelle Bédard, Carsten Bindslev‐Jensen, Anna Bedbrook, Sinthia Bosnic‐Anticevich, Luisa Brussino, Victoria Cardona, Alvaro A. Cruz, Govert de Vries, Philippe Devillier, Wytske J. Fokkens, José Miguel Fuentes‐Pérez, Bilun Gemicioğlu, Tari Haahtela, Yunen Rocío Huerta‐Villalobos, Juan Carlos Ivancevich, Inger Kull, Piotr Kuna, Violeta Kvedariene, Désirée E. Larenas Linnemann, Daniel Laune, Michael Makris, Erik Melén, Mário Morais‐Almeida, Ralph Mösges, Joaquim Mullol, Robyn E. O'Hehir, Nikolaos G. Papadopoulos, Ana Margarida Pereira, Emmanuel P. Prokopakis, Fotis Psarros, Frederico S. Regateiro, Sietze Reitsma, Boleslaw Samolinski, Nicola Scichilone, Jane da Silva, Cristiana Stellato, Ana Todo‐Bom, Peter Valentin Tomazic, Sanna Toppila Salmi, Antonio Valero, Arunas Valiulis, Erkka Valovirta, Michiel van Eerd, Maria Teresa Ventura, Arzu Yorgancioglu, Xavier Basagaña, Josep M. Antó, Jean Bousquet, João Almeida Fonseca

**Affiliations:** ^1^ MEDCIDS – Department of Community Medicine, Information and Health Decision Sciences Faculty of Medicine, University of Porto Porto Portugal; ^2^ CINTESIS – Center for Health Technology and Services Research University of Porto Porto Portugal; ^3^ RISE – Health Research Network University of Porto Porto Portugal; ^4^ Department of Computing Science Umeå University Umeå Sweden; ^5^ Department of Otorhinolaryngology, Head and Neck Surgery Section of Rhinology and Allergy University Hospital Marburg Philipps Universität Marburg Marburg Germany; ^6^ Department of Otolaryngology, Head and Neck Surgery Universitätsmedizin Mainz Mainz Germany; ^7^ Center for Rhinology and Allergology Wiesbaden Germany; ^8^ Department of Dermatology and Allergy Comprehensive Allergy Center Charité Universitätsmedizin Berlin Humboldt‐Universität zu Berlin Berlin Germany; ^9^ MASK‐air Montpellier France; ^10^ Medical Consulting Czarlewski Levallois France; ^11^ MACVIA‐France Montpellier France; ^12^ ISGlobAL Universitat Pompeu Fabra (UPF) CIBER Epidemiología y Salud Pública (CIBERESP) Barcelona Spain; ^13^ Equipe d’Epidémiologie Respiratoire Intégrative Université Paris‐Saclay UVSQ Université Paris‐Sud INSERM CESP Villejuif France; ^14^ Department of Dermatology and Allergy Centre Odense Research Center for Anaphylaxis (ORCA) Odense University Hospital Odense Denmark; ^15^ Quality Use of Respiratory Medicine Group Woolcock Institute of Medical Research The University of Sydney Sydney New South Wales Australia; ^16^ Sydney Local Health District Glebe New South Wales Australia; ^17^ Department of Medical Sciences Allergy and Clinical Immunology Unit University of Torino & Mauriziano Hospital Torino Italy; ^18^ Allergy Section Department of Internal Medicine Hospital Vall d’Hebron & ARADyAL research network Barcelona Spain; ^19^ Fundação ProAR – Federal University of Bahia Salvador Brazil; ^20^ WHO GARD Planning Group Salvador Brazil; ^21^ Peercode BV Geldermalsen The Netherlands; ^22^ Laboratoire de Pharmacologie UPRES EA220 Pôle des Maladies des Voies Respiratoires Hôpital Foch Université Paris‐Saclay Suresnes France; ^23^ Department of Otorhinolaryngology Amsterdam University Medical Centres Amsterdam the Netherlands; ^24^ Hospital General Regional IMSS General Regional Hospital 1 Dr. Carlos Mac Gregor Sanchez Navarro Mexico City Mexico; ^25^ Department of Pulmonary Diseases Istanbul University‐Cerrahpaşa Cerrahpasa Faculty of Medicine Istanbul Turkey; ^26^ Skin and Allergy Hospital Helsinki University Hospital Helsinki Finland; ^27^ Servicio de Alergia e Immunologia Clinica Santa Isabel Buenos Aires Argentina; ^28^ Department of Clinical Science and Education Södersjukhuset Karolinska Institutet Stockholm Sweden; ^29^ Division of Internal Medicine, Asthma and Allergy Barlicki University Hospital Medical University of Lodz Poland; ^30^ Department of Pathology Faculty of Medicine Institute of Biomedical Sciences Vilnius University Vilnius Lithuania; ^31^ Clinic of Chest Diseases and Allergology Faculty of Medicine Institute of Clinical Medicine Vilnius University Vilnius Lithuania; ^32^ Center of Excellence in Asthma and Allergy Hospital Médica Sur México City Mexico; ^33^ KYomed INNOV Montpellier France; ^34^ Allergy Unit “D Kalogeromitros” 2nd Dpt of Dermatology and Venereology National & Kapodistrian University of Athens “Attikon” University Hospital Athens Greece; ^35^ Sachs' Children and Youth Hospital Södersjukhuset Stockholm Sweden; ^36^ Institute of Environmental Medicine Karolinska Institutet Stockholm Sweden; ^37^ Allergy Center CUF Descobertas Hospital Lisbon Portugal; ^38^ Medical Faculty Institute of Medical Statistics, and Computational Biology University of Cologne Cologne Germany; ^39^ CRI‐Clinical Research International‐Ltd Hamburg Germany; ^40^ Rhinology Unit & Smell Clinic ENT Department Hospital Clínic Clinical & Experimental Respiratory Immunoallergy IDIBAPS University of Barcelona CIBERES Barcelona Spain; ^41^ Department of Allergy, Immunology and Respiratory Medicine Central Clinical School Monash University Melbourne Victoria Australia; ^42^ Alfred Health Melbourne Victoria Australia; ^43^ Center for Pediatrics and Child Health Institute of Human Development Royal Manchester Children's Hospital University of Manchester Manchester UK; ^44^ Allergy Department 2nd Pediatric Clinic Athens General Children's Hospital “P&A Kyriakou” University of Athens Athens Greece; ^45^ Department of Otorhinolaryngology University of Crete School of Medicine Heraklion Greece; ^46^ Allergy Department Athens Naval Hospital Athens Greece; ^47^ Allergy and Clinical Immunology Unit Institute of Immunology Faculty of Medicine Centro Hospitalar e Universitário de Coimbra University of Coimbra Coimbra Portugal; ^48^ Faculty of Medicine ICBR – Coimbra Institute for Clinical and Biomedical Research CIBB University of Coimbra Coimbra Portugal; ^49^ Department of Prevention of Environmental Hazards and Allergology Medical University of Warsaw Warsaw Poland; ^50^ PROMISE Department University of Palermo Palermo Italy; ^51^ Allergy Service Internal Medicine Department University Hospital of Federal University of Santa Catarina (HU‐UFSC) Florianópolis Brazil; ^52^ Department of Medicine, Surgery and Dentistry “Scuola Medica Salernitana” University of Salerno Salerno Italy; ^53^ Department of ENT Medical University of Graz Graz Austria; ^54^ Pneumology and Allergy Department CIBERES and Clinical & Experimental Respiratory Immunoallergy IDIBAPS University of Barcelona Spain; ^55^ Department of Public Health Vilnius University Institute of Clinical Medicine Clinic of Children's Diseases Institute of Health Sciences Vilnius Lithuania; ^56^ European Academy of Paediatrics (EAP/UEMS‐SP) Brussels Belgium; ^57^ Department of Lung Diseases and Clinical Immunology University of Turku Turku Finland; ^58^ Terveystalo Allergy Clinic Turku Finland; ^59^ Unit of Geriatric Immunoallergology University of Bari Medical School Bari Italy; ^60^ Department of Pulmonary Diseases Faculty of Medicine Celal Bayar University Manisa Turkey; ^61^ Universitat Pompeu Fabra (UPF) Barcelona Spain; ^62^ CIBER Epidemiología y Salud Pública (CIBERESP) Barcelona Spain; ^63^ IMIM (Hospital del Mar Research Institute) Barcelona Spain; ^64^ Centre Hospitalier Universitaire Montpellier France

**Keywords:** allergic rhinitis, mobile health, reliability, responsiveness, visual analog scales

## Abstract

**Background:**

MASK‐air® is an app that supports allergic rhinitis patients in disease control. Users register daily allergy symptoms and their impact on activities using visual analog scales (VASs). We aimed to assess the concurrent validity, reliability, and responsiveness of these daily VASs.

**Methods:**

Daily monitoring VAS data were assessed in MASK‐air® users with allergic rhinitis. Concurrent validity was assessed by correlating daily VAS values with those of the EuroQol‐5 Dimensions (EQ‐5D) VAS, the Control of Allergic Rhinitis and Asthma Test (CARAT) score, and the Work Productivity and Activity Impairment Allergic Specific (WPAI‐AS) Questionnaire (work and activity impairment scores). Intra‐rater reliability was assessed in users providing multiple daily VASs within the same day. Test–retest reliability was tested in clinically stable users, as defined by the EQ‐5D VAS, CARAT, or “VAS Work” (i.e., VAS assessing the impact of allergy on work). Responsiveness was determined in users with two consecutive measurements of EQ‐5D‐VAS or “VAS Work” indicating clinical change.

**Results:**

A total of 17,780 MASK‐air® users, with 317,176 VAS days, were assessed. Concurrent validity was moderate–high (Spearman correlation coefficient range: 0.437–0.716). Intra‐rater reliability intraclass correlation coefficients (ICCs) ranged between 0.870 (VAS assessing global allergy symptoms) and 0.937 (VAS assessing allergy symptoms on sleep). Test–retest reliability ICCs ranged between 0.604 and 0.878—“VAS Work” and “VAS asthma” presented the highest ICCs. Moderate/large responsiveness effect sizes were observed—the sleep VAS was associated with lower responsiveness, while the global allergy symptoms VAS demonstrated higher responsiveness.

**Conclusion:**

In MASK‐air®, daily monitoring VASs have high intra‐rater reliability and moderate–high validity, reliability, and responsiveness, pointing to a reliable measure of symptom loads.

## 1 INTRODUCTION

Allergic rhinitis is a burdensome condition contributing to a substantial loss of work and school productivity, as well as to decreased quality of life.^1^, ^2^ While there have been important advances on the treatment of allergic rhinitis, many patients remain poorly controlled.^3^ Mobile health‐based approaches may contribute to addressing this problem.^4^ As an example, MASK‐air® is a mobile app available in 25 countries, comprising a daily monitoring questionnaire assessing the impact that allergic symptoms have on the user each day.^3^, ^5^, ^6^, ^7^, ^8^, ^9^, ^10^

In MASK‐air®, visual analog scales (VASs) are used for several questions on daily monitoring. Such VASs range from “not at all bothersome” (0) to “extremely bothersome” (100) and indicate the degree to which nose, eye, or asthma symptoms bother users during the day (or specifically impact their work or sleep activities). While the concurrent validity of some of these VASs has already been assessed,^5^ it has not yet been established for all VASs, particularly when taking all validated comparators into account. In addition, their reliability and responsiveness have not yet been evaluated. In fact, reliability estimates are needed to provide information on the stability of measures/inputs obtained at different times of the day from the same users (intra‐rater reliability) or on different days in users considered clinically stable (test–retest reliability).^11^ On the other hand, responsiveness estimates inform on the ability of daily monitoring VASs to change over a specific period of time in cases where changes in a reference measure of health status have occurred.^12^

An assessment of such properties is essential for determining whether daily monitoring VASs can actually be used as a reliable tool for measuring rhinitis control. Therefore, this study aimed to assess concurrent validity, intra‐rater and test–retest reliability, as well as the responsiveness of MASK‐air® daily monitoring VASs.

## 2 METHODS

### 2.1 Study design

We assessed the concurrent validity, reliability, and responsiveness of each daily monitoring VAS of MASK‐air®. Reliability was evaluated by assessing intra‐rater reliability (assessing the agreement between multiple values provided by the same users within the same day) and test–retest reliability (assessing the agreement between different daily VAS results provided by clinically stable patients). The main analysis concerned all countries in which MASK‐air® is available. Sub‐analyses using only data from European users were also performed.

### 2.2 Setting and participants

MASK‐air® has been available since 2015. It is currently being used in 25 countries and 19 languages (www.mask‐air.com). We included the daily monitoring data of MASK‐air® users aged 16–90 years and with a self‐reported diagnosis of allergic rhinitis.

MASK‐air® is used by people who find it on the Internet (namely on Apple App store or Google Play). Some of the users are patients who were asked by their physicians to use it. However, due to privacy rules, it is impossible to determine how patients come to use the app.

### 2.3 Ethics

MASK‐air® is CE1‐registered but was not considered by the Ethical Committee of the Cologne Hospital (2017) as a medical device, given that it does not provide any recommendations concerning treatment or diagnosis. MASK‐air® follows GDPR regulations. An independent review board approval was not required for this specific study, as all data had been anonymized prior to the study (including geolocation‐related data) using k‐anonymity, and users agreed to have their data analyzed in the terms of use (translated into all languages and first customized according to the legislation of each country, allowing the use of the results for research purposes).^13^, ^14^

### 2.4 Data sources and variables

We analyzed the MASK‐air® daily monitoring data up to December 6, 2020. The daily monitoring of symptoms comprises six mandatory questions (addressing the period of 1 day), whose responses are provided by means of VASs:

- How much the overall allergic symptoms bothered the user on that day (“VAS global allergy symptoms”);
- How much nasal symptoms bothered the user on that day (“VAS nose”);
- How much ocular symptoms bothered the user on that day (“VAS eyes”);
- How much asthma symptoms bothered the user on that day (“VAS asthma”);
- How well the user slept on the previous night (“VAS sleep”); and
- How sleepy the user was during the day (“VAS sleepiness”).

In addition, if users report that they are working on that day, they are asked how much their allergic symptoms affected work activities on that day (“VAS work”).

When reporting daily VAS, users are asked to provide their daily medication using a scroll list customized for each country.^15^

When responding to the MASK‐air® daily monitoring questionnaire, it is not possible to skip any of the questions, precluding missing data. While symptoms should be monitored on a daily basis, some users may have provided more than one daily input.

In addition to the daily monitoring of symptoms, MASK‐air® users need to provide further clinical information (e.g., indicate their diagnosed allergies), and may respond to other questionnaires, including EuroQol‐5 Dimensions (EQ‐5D‐5L), the Control of Allergic Rhinitis and Asthma Test (CARAT), Work Productivity and Activity Impairment: Allergic Specific (WPAI:AS), and the Epworth Sleepiness Scale (ESS). MASK‐air® users can answer these questionnaires on any day they want to, and either before or after answering the daily monitoring questionnaire. EQ‐5D‐5L assesses the respondents’ health status through five dimensions/questions (each with five levels) followed by a VAS assessing the general health status on that day.^16^, ^17^ CARAT is a 10‐item questionnaire assessing the control of allergic rhinitis and asthma in the previous 4 weeks, with four questions specifically concerning nasal symptoms.^18^, ^19^ WPAI:AS is a 9‐item questionnaire assessing the weekly impact of allergies on work and academic productivity, with three of its questions allowing estimation of the overall work impairment due to allergy, and the ninth question assessing the overall activity impairment due to allergy.^20^, ^21^ Finally, ESS evaluates respondents’ sleepiness by assessing the chances of dozing in eight possible scenarios on that day.^22^, ^23^

### 2.5 Biases

Potential information biases were addressed by restricting our analyses to data from users with a self‐reported diagnosis of allergic rhinitis. Exclusion of data from users aged less than 16 years allowed us to address potential variability associated with age (i.e., differences between children and adults).

### 2.6 Sample size

We did not perform sample size calculation, but rather analyzed all data from users meeting the eligibility criteria and with valid data. Nevertheless, analyses were not performed for situations/comparators in which the number of users providing data for at least one daily monitoring VAS never exceeded 50.

### 2.7 Data analysis

Concurrent validity was assessed using statistical functions in Microsoft Excel 2016. All the other analyses were performed using software R (version 4.0, R Foundation for Statistical Computing, Vienna, Austria), with intraclass correlation coefficients (ICCs) being calculated with the “irr” package.^24^

#### 2.7.1 Concurrent validity

Concurrent validity was assessed by computing Spearman correlation coefficients for the associations between three daily monitoring VASs (“VAS global allergy symptoms,” “VAS Nose,” and “VAS work”) and EQ‐5D VAS, CARAT (considering CARAT as a whole, as well as just the first four questions of CARAT—which concern the upper airways—and just the last six questions) and WPAI:AS (considering the “percentage of overall work impairment due to allergy” and question number 9—“percentage of activity impairment due to allergy”). In addition, correlations between different daily monitoring VASs were computed. Confidence intervals were estimated with alpha at 0.001 (indicating a 99.9% confidence level) and with standard deviation being Fisher’s *z*‐transformation of the Spearman correlation coefficient. We considered coefficients of 0.5 to 0.8 (or −0.8 to −0.5) to indicate moderate correlation, and of >0.8 (or <−0.8) to indicate strong correlation.

#### 2.7.2 Assessment of intra‐rater reliability

The assessment of intra‐rater reliability for daily monitoring VASs was estimated considering users providing multiple inputs within the same day. For each day of use (of the same user) with multiple inputs for the same VAS, we computed the difference between the first and the second inputs for each VAS. We computed the average difference for each VAS, as well as the frequency of cases in which (i) such same‐day values were the same, (ii) the difference between such same‐day values did not exceed 10 units, and (iii) the difference between such same‐day values exceeded 10 units (differences lower than 10 units in MASK‐air® VAS point to low intra‐individual response variability^5^). We calculated the ICC for assessment of intra‐rater reliability (using two‐way models estimating absolute agreement, based on average measurements^25^, ^26^), also taking into account the first and second inputs within the same day by the same user. A sensitivity analysis was performed considering the first and last inputs for each VAS (instead of the first and second inputs).

#### 2.7.3 Assessment of test–retest reliability

We assessed test–retest reliability for each daily monitoring VAS. Assessment of test–retest reliability implies the identification of users with two measurements of a validated comparator indicating clinical stability. In this study, we used three different validated comparators for assessment of test–retest reliability: EQ‐5D VAS, CARAT, and “VAS work” (despite being a daily monitoring VAS, the validity of “VAS work” was demonstrated in previous studies as well as in the assessment of its concurrent validity with WPAI:AS). WPAI:AS (“percentage of overall work impairment due to allergy”) was also used to define clinical stability when assessing test–retest reliability for “VAS work.” On the other hand, the ESS was used to define clinical stability when assessing test–retest reliability for “VAS sleep” and “VAS sleepiness.”

Clinical stability was assumed whenever a user had two consecutive measurements less than 5 weeks apart, with results for validated comparators having a difference smaller than the minimal clinically important difference (MCID) value. Whenever the same user had more than two consecutive measurements (or more than one set of measurements) meeting the aforementioned criteria, the first two measurements were selected. Agreement was assessed by estimating ICCs using two‐way models estimating absolute agreement, based on average measurements.^25^, ^26^ We considered that ICCs of <0.5 indicate low reliability (both for test–retest reliability and intra‐rater reliability), those of 0.5–0.75 indicate moderate reliability, and those of >0.75 indicate high reliability.^25^

In the case of CARAT, differences ≤3 were considered to be lower than the MCID.^27^ For the remaining comparators, no MCID for patients with allergic rhinitis has been defined. Therefore, such values were determined based on distribution‐based methods—we considered the MCID to correspond to 0.5 × standard deviation of the baseline observations.^28^ Based on such an approach, we estimated an MCID of 10 points for EQ‐5D‐VAS, of 11 points for “VAS work,” and of 14% for WPAIS:AS. For ESS, we considered clinical stability if the same category was observed for the different daily measurements.^29^

#### 2.7.4 Responsiveness

We assessed responsiveness for each daily monitoring VAS. Assessment of responsiveness implies the identification of users with two measurements of a validated comparator indicating clinical change. In this study, validated comparators to indicate clinical change included the EQ‐5D VAS and “VAS work.” We were not able to use CARAT, WPAI:AS (to assess “VAS work” responsiveness), or ESS (to assess “VAS sleep” and “VAS sleepiness” responsiveness) as comparators, given that clinical change based on such measurements was observed in less than 50 users for every daily monitoring VAS.

Clinical change was assumed whenever a user had two consecutive measurements more than 5 weeks apart, with results for validated comparators having a difference equal to or higher than the MCID value. Following the impossibility of using CARAT (which assesses a period of 4 weeks) as a comparator, we performed a sub‐analysis defining the EQ‐5D VAS and “VAS work” clinical change based on periods more than 3 weeks apart. Whenever the same user had more than two consecutive measurements (or more than one set of measurements) meeting the aforementioned criteria, the first two inputs were selected.

Responsiveness was determined by calculating Cohen’s effect size and the standardized response mean (SRM).^12^ Cohen’s effect size was calculated by dividing the mean difference between daily monitoring VASs by the standard‐deviation of “baseline” VAS. The SRM was calculated by dividing the mean by the standard‐deviation of the differences between the daily monitoring VASs. For each of these measures, values of 0.2–0.5 were considered to represent small effect sizes, 0.5–0.8 medium effect sizes, and >0.8 large effect sizes.^12^

## 3 RESULTS

### 3.1 MASK‐air® data

**FIGURE 1:**
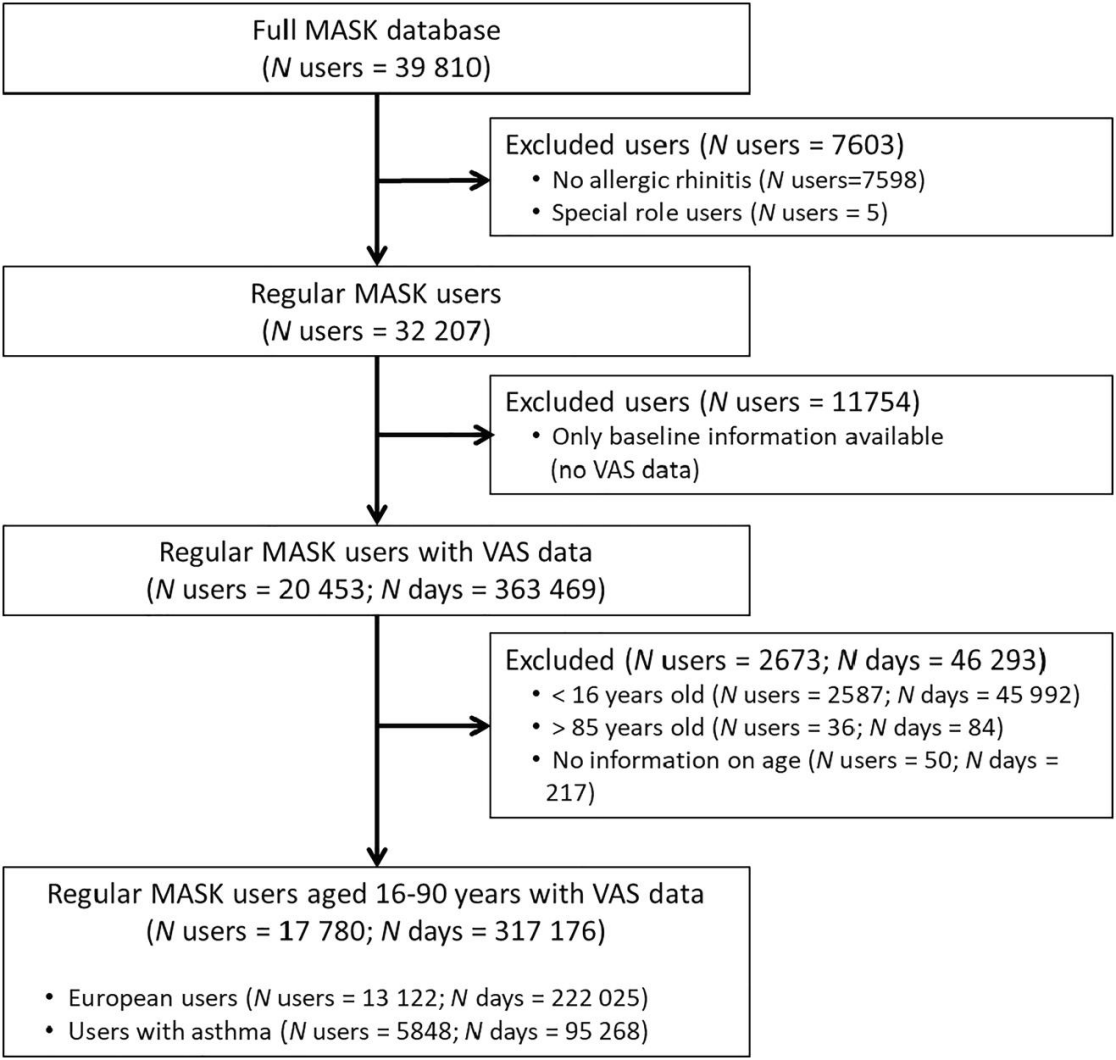
Flow diagram illustrating the selection of users/days of use meeting eligibility criteria

Up until December 6, 2020, 39,810 people had used MASK‐air®. Of these, 17,780 were aged 16–90 years, had self‐reported allergic rhinitis, and had responded at least once to the daily monitoring VAS (Figure 1). These 17,780 users provided daily monitoring VAS data for 317,176 days, corresponding to an average of 17.8 days per user.

Daily monitoring VAS median values ranged from 0 (“VAS asthma”) to 17 (“VAS sleep”) (Table 1). For the EQ‐5D VAS and CARAT, median values of 80 (interquartile range = 14) and 16 (interquartile range = 10) were respectively observed.

**TABLE 1:** Number of users and days of use for which there is information on daily monitoring visual analog scales (VASs) and on validated questionnaires in the MASK‐air® database

	*N* users	*N* days	Median value (Q1–Q3)
Daily monitoring VAS			
VAS global allergy symptoms^a^	17,780	317,154	11 (2–29)
VAS eyes^a^	17,780	317,176	4 (0–18)
VAS nose^a^	17,780	317,176	12 (2–30)
VAS asthma^a^	17,023	312,625	0 (0–10)
VAS work^a^	11,980	149,732	8 (0–23)
VAS sleep^a^	9009	151,011	17 (7–37)
VAS sleepiness^a^	8911	142,444	16 (6–35)
EQ‐5D VAS^b^	2478	4324	80 (66–90)
CARAT^c^	1086	2042	16 (11–21)
WPAI:AS^d^	1411	2308	15 (2–44)
ESS^e^	1081	2055	7 (4–11)

*Note*: The number of observations/days for each daily monitoring VAS is not always the same as, despite the impossibility of skipping questions when filling in each daily monitoring questionnaire, the MASK‐air® daily monitoring questionnaire has evolved over time, with some questions having been added later than others. On the other hand, VAS Work can only be filled in for days when users report to be working.

Abbreviations: CARAT, Control of Allergic Rhinitis and Asthma Test; ESS, Epworth, Sleepiness Scale; Q1–Q3, first quartile–third quartile; WPAI:AS, Work Productivity and Activity Impairment Allergic Specific (median and quartiles for “percent overall work impairment due to allergy”).

^a^ Scale ranging from 0 to 100, with higher values indicating worse status.

^b^ Scale ranging from 0 to 100, with higher values indicating better health status.

^c^ Scale ranging from 0 to 30, with higher values indicating better allergic rhinitis and asthma control.

^d^ Percent overall work impairment due to allergy—scale ranging from 0 to 100, with higher values indicating greater work impairment.

^e^ Scale ranging from 0 to 24, with higher values indicating greater daytime sleepiness.

### 3.2 Concurrent validity

Concurrent validity (Table 2) was calculated by mutually correlating nine different variables, including three daily monitoring VASs (“VAS global allergy symptoms,” “VAS nose,” and “VAS work”) and six comparators. For VAS global allergy symptoms, correlations varied between 0.437 [95% confidence interval (CI) = 0.411–0.462] (CARAT—questions 5–10) and 0.663 [95% CI = 0.637–0.688] (WPAI:AS—question 9). Similar results were observed for VAS nose. For “VAS work,” the strongest correlations were observed for WPAI:AS overall work impairment (0.716; 95% CI = 0.676–0.752) and for WPAI:AS question 9 (0.713; 95% CI = 0.675–0.747).

**TABLE 2:** Concurrent validity of visual analog scales (VASs) for global allergy symptoms (versus six comparators), nose symptoms and impact on work, using data from MASK‐air® users

Spearman correlation coefficient [95% CI] (*N* observations)	VAS global allergy symptoms	VAS nose	VAS work	EQ‐5D VAS	CARAT	CARAT (Q1–4)	CARAT (Q5–10)	WPAI:AS^a^	WPAI:AS (Q9)^b^
VAS global allergy symptoms	–	0.877 [0.875; 0.879] (317,154)	0.814 [0.811; 0.817] (149,726)	−0.512 [−0.533; −0.491] (4322)	−0.542^c^ [−0.571; −0.512] (2042)	−0.497 [−0.525; −0.469] (2042)	−0.437 [−0.462; −0.411] (2042)	0.623 [0.591; 0.653] (2306)	0.663 [0.637; 0.688] (3279)
VAS nose	–	–	0.773 [0.769; 0.776] (149,732)	−0.481 [−0.502; −0.461] (4324)	−0.523^c^ [−0.553; −0.492] (2042)	−0.530 [−0.561; −0.499] (2042)	−0.388 [−0.413; −0.362] (2042)	0.590 [0.559; 0.620] (2308)	0.613 [0.586; 0.638] (3281)
VAS work	–	–	–	−0.504 [−0.532; −0.475] (2324)	−0.359^c^ [−0.393; 0.324] (965)	−0.249 [−0.274; 0.224] (965)	−0.358 [−0.392; −0.323] (965)	0.716 [0.676; 0.752] (1461)	0.713 [0.675; 0.747] (1656)
EQ‐5D VAS	–	–	–	–	0.556 [0.515; 0.596] (1299)	0.371 [0.340; 0.401] (1299)	0.563 [0.522; 0.601] (1299)	−0.469 [−0.500; −0.438] (1783)	−0.489 [−0.515; −0.462] (2478)
CARAT	–	–	–	–	–	0.806 [0.776; 0.833] (2042)	0.882 [0.857; 0.902] (2042)	−0.559 [−0.613; −0.500] (641)	−0.599 [−0.643; −0.552] (1038)
CARAT (Q1–4)	–	–	–	–	–	–	0.451 [0.423; 0.479] (2042)	−0.350 [−0.392; −0.308] (641)	−0.443 [−0.481; −0.403] (1038)
CARAT (Q5–10)	–	–	–	–	–	–	–	−0.556 [−0.610; −0.497] (641)	−0.530 [−0.572; −0.485] (1038)
WPAI:AS	–	–	–	–	–	–	–	–	0.770 [0.681; 0.836] (314)
WPAI:AS (Q9)	–	–	–	–	–	–	–	–	–

Abbreviations: CARAT, Control of Allergic Rhinitis and Asthma Test; CI, confidence interval; *Q*, question; WPAI:AS, Work Productivity and Activity Impairment Allergy Specific.

^a^ Percent overall work impairment due to allergy.

^b^ Question 9 of WPAI:AS assesses overall activity impairment due to allergy.

^c^ Spearman correlation coefficients when considering the average VAS values for the 4 weeks prior to answering CARAT: −0.572 for the correlation between CARAT and VAS Global allergy symptoms; −0.559 for the correlation between CARAT and VAS Nose; and −0.466 for the correlation between CARAT and VAS Work.

### 3.3 Intra‐rater reliability

Between 2412 (“VAS work”) and 5827 (“VAS nose” and “VAS eyes”) days with more than one daily monitoring VAS input provided by the same user were recorded. For all VASs, more than 50% of the days had no differences in the first and second values provided within the same day (Table 3). Differences between the first and second daily values differing by more than 10 units ranged between 11.2% (“VAS asthma”) and 24.4% (“VAS nose”). ICCs varied between 0.870 (“VAS global allergy symptoms”) and 0.937 (“VAS sleep”). Similar results were observed when analyzing data from MASK‐air® European users, or when taking into account the first and last daily measurements (Tables 3 and 4).

**TABLE 3:** Results of intra‐rater reliability for daily monitoring visual analog scales (VASs) using data from MASK‐air® users where MASK‐air® is available

	*N* days with more than one daily value^a^	Mean difference between first and second daily values (SD)	*N* (%) observations with no difference between first and second daily values	*N* (%) observations with difference between first and second daily values >10 VAS units^a^	ICC
A. Data of users from all countries where MASK‐air® is available					
VAS global allergy symptoms^b^	5823 [1.8]	1.6 (17.3)	2809 (53.2)	994 (18.8) [0.3]	0.870 [0.863–0.877]
VAS eyes^b^	5827 [1.8]	1.0 (14.5)	3918 (67.2)	1002 (17.2) [0.3]	0.894 [0.889–0.900]
VAS nose^b^	5827 [1.8]	2.0 (16.8)	3219 (55.2)	1421 (24.4) [0.4]	0.890 [0.883–0.897]
VAS asthma^b^	5700 [1.8]	0.7 (12.2)	4365 (76.6)	636 (11.2) [0.2]	0.906 [0.901–0.911]
VAS work^b^	2412 [1.6]	1.4 (13.3)	1607 (66.7)	443 (17.3) [0.3]	0.902 [0.894–0.910]
VAS sleep^b^	2732 [1.8]	0.6 (12.1)	1966 (72.0)	359 (13.1) [0.2]	0.937 [0.932–0.942]
VAS sleepiness^b^	2567 [1.8]	1.3 (12.2)	1838 (71.6)	367 (14.3) [0.3]	0.931 [0.925–0.936]
B. Data of European MASK‐air® users					
VAS global allergy symptoms^b^	3546 [1.6]	1.8 (17.4)	1571 (44.3)	1048 (29.6) [0.5]	0.871 [0.862–0.880]
VAS eyes^b^	3550 [1.6]	1.4 (14.5)	2221 (62.5)	686 (19.3) [0.3]	0.903 [0.896–0.910]
VAS nose^b^	3550 [1.6]	2.3 (16.7)	1849 (52.1)	923 (26.0) [0.4]	0.892 [0.883–0.901]
VAS asthma^b^	3423 [1.6]	1.0 (13.1)	2492 (72.8)	457 (13.4) [0.2]	0.904 [0.897–0.910]
VAS work^b^	1415 [1.3]	1.6 (13.4)	868 (61.3)	284 (19.0) [0.3]	0.903 [0.892–0.913]
VAS sleep^b^	1666 [1.6]	0.6 (10.5)	1193 (71.6)	212 (12.7) [0.2]	0.952 [0.947–0.956]
VAS sleepiness^b^	1588 [1.6]	1.3 (12.4)	1096 (69.0)	251 (15.8) [0.2]	0.928 [0.920–0.935]

Abbreviations: ICC, intraclass correlation coefficient; SD, standard deviation.

^a^ Values in square brackets correspond to the percentage of such observations in relation to the total number of available observations for each VAS.

^b^ Scale ranging from 0 to 100, with higher values indicating worse status.

**TABLE 4:** Results of intra‐rater reliability for daily monitoring visual analog scales (VASs) using the first and last daily inputs

	*N* days with more than one daily value^a^	Mean difference between first and last daily values (SD)	*N* (%) observations with no difference between first and last daily values	*N* (%) observations with difference between first and last daily values >10 VAS units^a^	ICC
VAS global allergy symptoms^a^	5823 [1.8]	1.7 (18.0)	2719 (46.7)	1615 (27.7) [0.5]	0.859 [0.851–0.867]
VAS eyes^a^	5827 [1.8]	1.0 (15.0)	3835 (65.8)	1042 (17.9) [0.3]	0.886 [0.880–0.892]
VAS nose^a^	5827 [1.8]	2.0 (17.4)	3140 (53.9)	1472 (25.3) [0.5]	0.881 [0.873–0.888]
VAS asthma^a^	5700 [1.8]	0.7 (12.6)	4305 (75.5)	625 (11.0) [0.2]	0.900 [0.895–0.905]
VAS work^a^	2412 [1.6]	1.4 (14.0)	1569 (65.0)	443 (18.4) [0.3]	0.892 [0.883–0.901]
VAS sleep^a^	2732 [1.8]	0.8 (12.3)	1940 (71.0)	360 (13.3) [0.2]	0.935 [0.930–0.940]
VAS sleepiness^a^	2567 [1.8]	1.4 (12.4)	1810 (70.5)	368 (14.5) [0.3]	0.929 [0.923–0.934]

Abbreviations: ICC, intraclass correlation coefficient; SD, standard deviation.

^a^ Values in square brackets correspond to the percentage of such observations in relation to the total number of available observations for each VAS.

^b^ Scale ranging from 0 to 100, with higher values indicating worse status.

### 3.4 Test–retest reliability

Using clinical stability defined according to EQ‐5D VAS, the test–retest reliability of daily monitoring VASs was based on data from 102 (“VAS work”) to 270 (“VAS global allergy symptoms,” “VAS nose,” “VAS eyes,” and “VAS asthma”) users. The lowest ICC concerned “VAS sleep” (ICC = 0.675; 95% CI = 0.527–0.777) and “VAS sleepiness” (ICC = 0.686; 95% CI = 0.544–0.784), while the highest concerned “VAS work” (ICC = 0.823; 95% CI = 0.738–0.880) and “VAS asthma” (ICC = 0.857; 95% CI = 0.818–0.887) (Table 5).

**TABLE 5:** Results for test–retest analysis of the different daily monitoring visual analog scales (VASs) for MASK‐air® users from the 25 countries where MASK‐air® is available

	EQ‐5D VAS		CARAT		Work VAS	
	*N* users	ICC [95% CI]	*N* users	ICC [95% CI]	*N* users	ICC [95% CI]
A. Data of users from all countries where MASK‐air® is available						
VAS global allergy symptoms^a^	270	0.753 [0.662–0.817]	134	0.745 [0.637–0.821]	5761	0.848 [0.831–0.862]
VAS nose^a^	270	0.772 [0.708–0.821]	134	0.770 [0.668–0.839]	5763	0.841 [0.829–0.852]
VAS eyes^a^	270	0.747 [0.677–0.802]	134	0.740 [0.633–0.816]	5763	0.831 [0.822–0.840]
VAS asthma^a^	270	0.857 [0.818–0.887]	134	0.840 [0.775–0.886]	5763	0.878 [0.871–0.884]
VAS work^a^ ^,^ ^b^	102	0.823 [0.738–0.880]	35	0.823 [0.605–0.915]	–	–
VAS sleep^a^ ^,^ ^c^ ^,^ ^d^	110	0.675 [0.527–0.777]	112	0.604 [0.425–0.727]	2257	0.685 [0.658–0.711]
VAS sleepiness^a^ ^,^ ^c^ ^,^ ^d^	112	0.686 [0.544–0.784]	111	0.550 [0.344–0.692]	2078	0.748 [0.723–0.770]
B. Data of European MASK‐air® users						
VAS global allergy symptoms^a^	217	0.742 [0.636–0.814]	103	0.792 [0.692–0.859]	4115	0.855 [0.839–0.869]
VAS nose^a^	217	0.760 [0.683–0.818]	103	0.804 [0.709–0.867]	4117	0.842 [0.829–0.853]
VAS eyes^a^	217	0.711 [0.620–0.780]	103	0.748 [0.628–0.829]	4117	0.839 [0.829–0.849]
VAS asthma^a^	217	0.856 [0.812–0.890]	103	0.858 [0.791–0.904]	4117	0.875 [0.867–0.882]
VAS work^a^ ^,^ ^e^	83	0.827 [0.734–0.888]	31	0.809 [0.533–0.915]	–	–
VAS sleep^a^ ^,^ ^f^ ^,^ ^g^	92	0.649 [0.470–0.768]	87	0.616 [0.412–0.750]	1591	0.686 [0.653–0.715]
VAS sleepiness^a^ ^,^ ^f^ ^,^ ^g^	95	0.677 [0.515–0.784]	87	0.561 [0.332–0.713]	1507	0.739 [0.709–0.766]

Abbreviations: CARAT, Control of Allergic Rhinitis and Asthma Test; CI, confidence interval; ICC, intraclass correlation coefficient.

^a^ Scale ranging from 0 to 100, with higher values indicating worse status.

^b^ ICC for VAS work in clinically stable patients as defined by the percent overall work impairment of Work Productivity and Activity Impairment Allergic Specific = 0.749 [95% CI = 0.594–0.845] (*N* = 68 users).

^c^ ICC for VAS sleep in clinically stable patients as defined by the Epworth Sleepiness Scale = 0.620 [95% CI = 0.424–0.749] (*N* = 91 users).

^d^ ICC for VAS day Sleepiness in clinically stable patients as defined by the Epworth Sleepiness Scale = 0.627 [95% CI = 0.435–0.753] (*N* = 91 users).

^e^ ICC for VAS work in clinically stable patients as defined by the percent overall work impairment of the Work Productivity and Activity Impairment Allergic Specific = 0.857 [95% CI = 0.732–0.921] (*N* = 51 users).

^f^ ICC for VAS sleep in clinically stable patients as defined by the Epworth Sleepiness Scale = 0.647 [95% CI = 0.441–0.777] (*N* = 76 users).

^g^ ICC for VAS day sleepiness in clinically stable patients as defined by the Epworth Sleepiness Scale = 0.667 [95% CI = 0.471–0.790] (*N* = 75 users).

Using clinical stability defined according to CARAT, the test–retest reliability of daily monitoring VASs was based on data from up to 134 users. The lowest ICC concerned “VAS sleepiness” (ICC = 0.550; 95% CI = 0.344–0.692) and “VAS sleep” (ICC = 0.604; 95% CI = 0.425–0.727), while the highest concerned “VAS work” (ICC = 0.823; 95% CI = 0.605–0.915) and “VAS asthma” (ICC = 0.840; 95% CI = 0.775–0.886) (Table 5).

Using “VAS work” to define clinical stability, the test–retest reliability of daily monitoring VASs was based on data from 2078 (“VAS sleepiness”) to 5763 (“VAS nose,” “VAS eyes,” and “VAS global allergy symptoms”) users. As with the two previous scenarios, the lowest ICC was observed for VAS sleep (ICC = 0.685; 95% CI = 0.658–0.711) and VAS sleepiness (ICC = 0.748; 95% CI = 0.723–0.770), while the highest ICC was observed for VAS asthma (ICC = 0.878; 95% CI = 0.871–0.884) (Table 5).

Similar results were observed when analyzing data from MASK‐air European users (Table 5).

### 3.5 Responsiveness analysis

Using the EQ‐5D VAS to define clinical change (based on observations at least 5 weeks apart), we assessed the responsiveness of daily monitoring VASs based on data from up to 85 users (“VAS global allergy symptoms,” “VAS nose,” “VAS eyes,” and “VAS asthma”). Moderate effect sizes were observed for “VAS global allergy symptoms” (Cohen’s effect size = 0.572; SRM = 0.530), “VAS sleepiness” (Cohen’s effect size = 0.717; SRM = 0.566), and “VAS work” (Cohen’s effect size = 0.735; SRM = 0.719) (Table 6). For the remaining daily monitoring VASs, estimated effect sizes were lower than 0.5 (i.e., small effect sizes).

**TABLE 6:** Results for responsiveness analysis of the different daily monitoring visual analog scales (VASs) for MASK‐air® users from the 25 countries where MASK‐air® is available

	EQ‐5D VAS			VAS work		
	*N* users	Cohen ES	SRM	*N* users	Cohen ES	SRM
A. Observations at least 5 weeks apart						
VAS global allergy symptoms^a^	85	0.572	0.530	1156	0.994	0.931
VAS nose^a^	85	0.448	0.380	1157	0.877	0.781
VAS eyes^a^	85	0.417	0.411	1157	0.711	0.611
VAS asthma^a^	85	0.366	0.413	1157	0.573	0.512
VAS work^a^	29	0.735	0.719	–	–	–
VAS sleep^a^	27	0.386	0.411	484	0.482	0.405
VAS sleepiness^a^	28	0.717	0.566	450	0.625	0.554
B. Observations at least 3 weeks apart						
VAS global allergy symptoms^a^	108	0.467	0.451	1515	0.992	0.954
VAS nose^a^	108	0.423	0.379	1517	0.893	0.810
VAS eyes^a^	108	0.282	0.282	1517	0.694	0.598
VAS asthma^a^	108	0.328	0.351	1517	0.578	0.513
VAS work^a^	39	0.524	0.575	–	–	–
VAS sleep^a^	37	0.345	0.399	659	0.489	0.418
VAS sleepiness^a^	38	0.474	0.365	617	0.577	0.514

Abbreviations: ES, effect size; SRM, standardized response mean.

^a^ Scale ranging from 0 to 100, with higher values indicating worse status.

Using “VAS work” to define clinical change, we assessed responsiveness based on data from between 450 (“VAS sleepiness”) and 1157 (“VAS nose,” “VAS eyes,” and “VAS asthma”) users. We observed moderate effect sizes for “VAS asthma” (Cohen’s effect size = 0.573; SRM = 0.512), “VAS sleepiness” (Cohen’s effect size = 0.625; SRM = 0.554) and “VAS eyes” (Cohen’s effect size = 0.711; SRM = 0.611), and large effect sizes for “VAS nose” (Cohen’s effect size = 0.877; SRM = 0.781) and “VAS global allergy symptoms” (Cohen’s effect size = 0.994; SRM = 0.931) (Table 6).

When defining clinical change based on a period more than 3 weeks apart, similar results were observed when using “VAS work” as the comparator to define clinical change, while overall lower effect sizes were observed with EQ‐5D as a comparator (Table 6).

## 4 DISCUSSION

In this study, we observed that, overall, daily monitoring VASs presented with high intra‐rater reliability and moderate‐high concurrent validity, test–retest reliability and responsiveness. This is particularly relevant when taking into account the fact that, overall, the VAS has been shown to be a simple and sensitive instrument for measuring allergic rhinitis symptoms, having been used in both randomized controlled trials and observational studies.^30^ The incorporation of VASs into a mobile app and the demonstration of their reliability and responsiveness supports their use as a tool to guide users in controlling disease activity and adapting medication. Previous studies have already assessed other properties of daily monitoring VASs, observing strong correlation between “VAS work” and other VASs (namely “VAS global allergy symptoms,” “VAS nose,” “VAS eyes,” and “VAS asthma”).^5^, ^31^

The high intra‐rater reliability observed across the different daily monitoring VASs suggests that values provided within the same day for such scales do not tend to be substantially different. This is further supported by the consistency of results observed when the first and last daily inputs are taken into account (instead of the first and second ones).

On the other hand, test–retest analysis results indicate that daily monitoring VASs remain reasonably stable when clinical stability is attested by other validated comparators (EQ‐5D VAS, “VAS work” and CARAT). Overall, the presented ICCs are not dissimilar to those estimating test–retest reliability within the context of other measurement tools used in patients with rhinitis. For the Rhinitis Control Assessment Test, an ICC of 0.78 was observed,^32^ while for CARAT, the ICC was of 0.82.^19^ The lower ICC observed for sleep‐related VAS suggests that several other factors can potentially affect sleep, that the question is too simple (simply asking whether the user had slept well in the previous night or felt sleepy during the day), and/or that these VASs are not associated with the severity of allergy. However, there are several studies suggesting that sleep is impaired by rhinitis.^33^, ^34^, ^35^, ^36^ “VAS sleep” was also found to be associated with lower responsiveness. Responsiveness measures the occurrence of change (in this case, of daily monitoring VASs) in cases where clinical change is attested by other validated comparators.^12^ Responsiveness is typically assessed by effect size measures reporting on the magnitude of variation in relation to baseline or between‐subject variability, with higher values indicating larger changes.^12^ Therefore, in this study, we observed that daily monitoring VASs more strongly accompanied clinically relevant changes in “VAS work” than clinically relevant changes in EQ‐5D VAS. This may be related to the fact that the latter is not specific to allergic diseases. Differences of methods used to assess responsiveness impair comparisons with other tools used in rhinitis patients, such as the Rhinitis Control Assessment Test,^32^ CARAT,^19^ WPAI:AS,^20^ and ARIA‐C.^37^

This study has important limitations that are worth noting. Firstly, for some analyses (e.g., assessment of responsiveness in relation to the EQ‐5D VAS and CARAT), the number of users/days meeting the required conditions was relatively small (although not too dissimilar to the sample sizes used in the assessment of test–retest reliability in CARAT^19^ and in some groups of Rhinitis Control Assessment Test^32^), negatively affecting estimate precision. Such small numbers are explained not only by the conditions required to assess test–retest reliability and responsiveness, but also by the fact that, in MASK‐air®, CARAT, EQ‐5D, WPAI:AS, and ESS are not mandatory questionnaires within the context of daily monitoring. On the other hand, for each analysis, the number of users/days of use is not the same for each daily monitoring VAS. This is explained by the later introduction of certain VASs (namely “VAS sleep” and “VAS sleepiness”) in the daily monitoring questionnaire, as well as by the fact that “VAS work” is only answered on days when users report to be working.

Intra‐rater reliability was assessed based on different same‐day questionnaires by the same patient. However, within the same day (e.g., during the pollen season), a patient may experience changes in his/her symptoms. This potential limitation, however, results in an underestimation of intra‐rater reliability. That is, real intra‐rater reliability values may possibly be even higher than those we obtained. On the other hand, we used consecutive measurements less than 5 weeks apart in the definition of clinical stability. We cannot, however, exclude the possibility of “unstable periods” (i.e., clinically relevant changes) between these measurements. Nevertheless, the magnitude of such phenomenon is not expected to be particularly high, on account of the observed similarities in the test–retest ICC calculated based on CARAT (which assesses the previous month) and EQ‐5D‐VAS or “VAS work” (which assess a single day). Another important limitation concerns the main validated comparators used to assess concurrent validity, test–retest reliability and responsiveness. In fact, the EQ‐5D VAS is not specific for allergic diseases, measuring instead how good or bad the health of the respondent is on that day. On the other hand, while CARAT is specific for asthma and allergic rhinitis, it assesses allergic rhinitis (and asthma) control within the period of the last 4 weeks,^38^ while only one single day (the day being assessed) is contemplated in daily monitoring VASs. In addition, we were not able to use it as a comparator when assessing responsiveness, due to an insufficient sample size.

Furthermore, no MCID for allergic rhinitis patients had been previously defined for the EQ‐5D VAS and for “VAS Work.” We determined the corresponding MCID values within the context of this study, based on distribution‐based methods instead of anchor‐based methods. While the latter are often preferable, they imply the existence of an anchor‐based estimate,^39^ which was unavailable in this context (i.e., there was no “gold‐standard” variable to which the EQ‐5D VAS or “VAS work” could be compared accurately).

Finally, a selection bias may also be present on account of the representativeness of users and of days on which daily monitoring questionnaires were completed. In fact, it is expected that MASK‐air® users may not be representative of allergic rhinitis patients—only 5% of the daily monitoring data concerned users aged more than 65 years, and only 7% concerned current smokers. This suggests that MASK‐air® users are probably younger and more concerned about their health than overall allergic rhinitis patients. It is possible, however, that MASK‐air® users are representative of those allergic rhinitis patients using apps in the management of their disease. On the other hand, and despite the low median values of daily monitoring VASs, daily monitoring may more often be performed when users feel bothered about their allergic symptoms. In other words, regarding the days on which daily monitoring VASs were used, there may be an overrepresentation of “more troublesome” days. This is all the more relevant taking into account that, in this study, the mean adherence to MASK‐air® was found to be of 2.9% (with adherence/intensity of use calculated as the number of actual reporting days divided by the reporting period–following the methods of Di Fraia et al.^40^—computed as the period between December 6, 2020 and the date the user first used MASK‐air®). While this low adherence may be motivated by the fact that, for most users, MASK‐air® was not prescribed and promoted by their allergists, future studies should assess whether adherence patterns may have an impact on the validity and reliability of MASK‐air® VASs.

This study also has important strengths. We analyzed real‐world data from a diverse set of users with allergic rhinitis in 25 different countries and 19 languages. The structure of the app precluded the existence of missing data within each response to the MASK‐air® daily questionnaire. The validity, reliability and responsiveness of several VASs were assessed, with these VASs reflecting different types of allergic symptoms and different ways by which such symptoms can impact on users’ activities. In our analyses, we used three different main validated comparators (along with ESS for sleep‐related daily monitoring VASs), with consistent results being mostly obtained when identifying the best‐performing and worst‐performing daily monitoring VASs across the different analyses performed with different comparators. Finally, our results were robust to different sub‐analyses or sensitivity analyses performed: similar results were observed when analyzing data restricted to European users or, for intra‐rater reliability, when considering the first and last daily measurements (instead of the first and second ones).

In conclusion, in this study, we observed that daily monitoring VASs present high intra‐rater reliability and moderate‐high concurrent validity and test–retest reliability, with responsiveness being more variable across the different daily monitoring VASs and according to the chosen comparator. These results indicate that daily monitoring VASs are accurate instruments for measuring the daily impact of allergic rhinitis, possibly providing support for adapting medication and for controlling disease activity. Future research may focus on improving already existing scores or on developing new tools: (i) assessing daily rhinitis control, (ii) combining results from different daily monitoring VASs, (iii) including information on medication use (that can be obtained using MASK‐air®), and (iv) providing information on patients’ demographic and clinical characteristics.

## CONFLICTS OF INTERESTS

Sinthia Bosnic‐Anticevich reports grants from TEVA and personal fees from TEVA, AstraZeneca, Boehringer Ingelheim, GSK, Sanofi, Mylan. Jean Bousquet reports personal fees from Chiesi, Cipla, Hikma, Menarini, Mundipharma, Mylan, Novartis, Sanofi‐Aventis, Takeda, Teva, Uriach, other from KYomed‐Innov, and personal fees from Purina. Victoria Cardona reports personal fees from ALK, Allergy Therapeutics, LETI, Thermofisher, Merck, Astrazeneca, and GSK. Alvaro A Cruz reports grants and personal fees from GlaxoSmithKline, personal fees from Sanofi, AstraZeneca, Novartis, Chiesi, Boehringer Ingelheim, Mylan, and Eurofarma. Philippe Devillier reports personal fees from Mylan. Bilun Gemicioğlu reports grants from AstraZeneca, MSD, GSK, Deva, Novartis, and Abdi Ibrahim. Tari Haahtela reports personal fees from GSK, Mundipharma, Orion Pharma, Sanofi, Juan Carlos Ivancevich reports personal fees from Sanofi, Faes Farma, Laboratorios Casasco, Abbott, Ludger Klimek reports grants and personal fees from Allergopharma, LETI Pharma, MEDA/Mylan, and Sanofi, personal fees from Allergy Therapeut, Casssela med, and HAL Allergie, grants from ALK Abelló, Stallergenes, Quintiles, ASIT biotech, Lofarma, AstraZeneca, GSK, Inmunotk, and Membership: AeDA, DGHNO, Deutsche Akademie für Allergologie und klinische Immunologie, HNO‐BV, GPA, and EAACI. Piotr Kuna reports personal fees from Adamed, AstraZeneca, Berlin Chemie Menarini, Boehringer Ingelheim, GSK, HAL Allergy, Lekam, Mylan, Novartis, and Teva. Désirée Larenas Linnemann reports personal fees from Allakos, Amstrong, Astrazeneca, DBV Technologies, Grunenthal, GSK, Mylan, Menarini, MSD, Novartis, Pfizer, Sanofi, Siegfried, UCB, Alakos, and Gossamer, grants from Sanofi, Astrazeneca, Novartis, Circassia, UCB, GSK, TEVA, and Purina Institute. Michael Makris reports personal fees from Novartis, AstraZeneca, GSK, Chiesi, Menarini, Sanofi Genzyme, and Pfizer. Joaquin Mullol reports personal fees and other from SANOFI‐GENZYME & REGENERON, Genentech‐Roche & NOVARTIS, grants and personal fees from VIATRIS (MEDA‐MYLAN Pharma) and URIACH Group, and personal fees from Mitsubishi‐Tanabe, Menarini, UCB, AstraZeneca, and GSK. Oliver Pfaar reports grants and personal fees from ALK‐Abelló, Allergopharma, Stallergenes Greer, HAL Allergy Holding B.V./HAL Allergie GmbH, Bencard Allergie GmbH/Allergy Therapeutics, Lofarma, Anergis S.A., ASIT Biotech Tools S.A., Laboratorios LETI/LETI Pharma, and Glaxo Smith Kline, grants from Biomay, Circassia, Pohl‐Boskamp, and Inmunotek S.L., and personal fees from MEDA Pharma/MYLAN, Mobile Chamber Experts (a GA^2^LEN Partner), Indoor Biotechnologies, Astellas Pharma Global, EUFOREA, ROXALL Medizin, Novartis, Sanofi‐Aventis and Sanofi‐Genzyme, Med Update Europe GmbH, streamedup! GmbH, John Wiley and Sons, AS, and Paul‐Martini‐Stiftung (PMS). Nikolaos G. Papadopoulos reports personal fees from Novartis, Nutricia, HAL, MENARINI/FAES FARMA, SANOFI, MYLAN/MEDA, BIOMAY, AstraZeneca, GSK, MSD, ASIT BIOTECH, and Boehringer Ingelheim and grants from Gerolymatos International SA, and Capricare. Boleslaw Samolinski reports personal fees from Allergopharma, Polpharma, Viatris, TEVA, ADAMED, patient ombudsman, and Polish Allergology Society, grants from AstraZeneca, National Health Programme, and grants and personal fees from AstraZeneca. Ana Todo‐Bom reports grants and personal fees from AstraZeneca, GSK (GlaxoSmithKline), Novartis, Sanofi, Teva, and Mundipharma, personal fees from Bial, and grants from Leti. Torsten Zuberbier reports personal fees from Bayer Health Care, FAES, Novartis, Henkel, Novartis, from Henkel, AstraZeneca, AbbVie Fee for talk, ALK Fee for talk, Almirall Fee for talk, Astellas Fee for talk, Bayer Health Care Fee for talk, Bencard Fee for talk, Berlin Chemie Fee for talk, FAES Fee for talk, HAL Fee for talk, Leti Fee for talk, Meda Fee for talk, Menarini Fee for talk, Merck Fee for talk, MSD Fee for talk, Novartis Fee for talk, Pfizer Fee for talk, Sanofi Fee for talk, Stallergenes Fee for talk, Takeda Fee for talk, Teva Fee for talk, UCB Fee for talk, Henkel Fee for talk, Kryolan Fee for talk, and L'Oréal Fee for talk.

## AUTHOR CONTRIBUTIONS

Jean Bousquet: writing‐review & editing (equal); Bernardo Sousa Pinto: conceptualization (equal); Patrik Eklund: conceptualization (equal); Oliver Pfaar: conceptualization (equal); Ludger Klimek: conceptualization (equal); Torsten Zuberbier: conceptualization (equal); Wienczylslawa Czarlewski: conceptualization (equal); Annabelle Bedard: conceptualization (equal); Carsten Bindslev‐Jensen: conceptualization (equal); Anna Bedbrook: Supervision (equal); Sinthia Bosnic‐Anticevich: conceptualization (equal); Luisa Brussino: conceptualization (equal); Victòria Cardona: conceptualization (equal); Alvaro Cruz: conceptualization (equal); Govert De Vries: conceptualization (equal); Philippe Devillier: conceptualization (equal); Wytske Fokkens: conceptualization (equal); José Miguel Fuentes Perez: conceptualization (equal); Bilun Gemicioglu: conceptualization (equal); Tari Haahtela: conceptualization (equal); Yunen Rocio Huerta Villalobos: conceptualization (equal); Juan Carlos Ivancevich: conceptualization (equal); Inger Kull: conceptualization (equal); Piotr Kuna: conceptualization (equal); Violeta Kvedariene: conceptualization (equal); Desiree Larenas‐Linnemann: conceptualization (equal); Daniel Laune: conceptualization (equal); Michael Makris: conceptualization (equal); Erik Melen: conceptualization (equal); Mario Morais Almeida: conceptualization (equal); Ralph Mösges: conceptualization (equal); Joaquim Mullol: conceptualization (equal); Robyn O'Hehir: conceptualization (equal); Nikolaos Papadopoulos: conceptualization (equal); Ana Pereira: conceptualization (equal); Emmanuel Prokopakis: conceptualization (equal); Fotis Psarros: conceptualization (equal); Frederico Regateiro: conceptualization (equal); Sietze Reitsma: conceptualization (equal); Boleslaw Samolinski: conceptualization (equal); Nicola Scichilone: conceptualization (equal); Jane Da Silva: conceptualization (equal); Cristiana Stellato: conceptualization (equal); Ana Todo Bom: conceptualization (equal); Peter Tomazic: conceptualization (equal); Sanna Toppila Salmi: conceptualization (equal); Antonio Valero: conceptualization (equal); Arunas Valiulis: conceptualization (equal); Erkka Valovirta: conceptualization (equal); Michiel van Eerd: conceptualization (equal); Maria Teresa Ventura: conceptualization (equal); Arzu Yorgancioglu: conceptualization (equal); Xavier Basagana: conceptualization (equal); Josep Anto: conceptualization (equal); João Almeida Fonseca: conceptualization (equal).
